# Translational Regulation of Utrophin by miRNAs

**DOI:** 10.1371/journal.pone.0029376

**Published:** 2011-12-27

**Authors:** Utpal Basu, Olga Lozynska, Catherine Moorwood, Gopal Patel, Steve D. Wilton, Tejvir S. Khurana

**Affiliations:** 1 Department of Physiology, Pennsylvania Muscle Institute, School of Medicine, University of Pennsylvania, Philadelphia, Pennsylvania, United States of America; 2 Centre for Neuromuscular and Neurological Disorders, University of Western Australia, Perth, Australia; Southern Illinois University School of Medicine, United States of America

## Abstract

**Background:**

Utrophin is the autosomal homolog of dystrophin, the product of the Duchenne Muscular Dystrophy (DMD) locus. Its regulation is of therapeutic interest as its overexpression can compensate for dystrophin's absence in animal models of DMD. The tissue distribution and transcriptional regulation of utrophin have been characterized extensively, and more recently translational control mechanisms that may underlie its complex expression patterns have begun to be identified.

**Methodology/Principal Findings:**

Using a variety of bioinformatic, molecular and cell biology techniques, we show that the muscle isoform utrophin-A is predominantly suppressed at the translational level in C2C12 myoblasts. The extent of translational inhibition is estimated to be ∼99% in C2C12 cells and is mediated by both the 5′- and 3′-UTRs of the utrophin-A mRNA. In this study we identify five miRNAs (let-7c, miR-150, miR-196b, miR-296-5p, miR-133b) that mediate the repression, and confirm repression by the previously identified miR-206. We demonstrate that this translational repression can be overcome by blocking the actions of miRNAs, resulting in an increased level of utrophin protein in C2C12 cells.

**Conclusions/Significance:**

The present study has identified key inhibitory mechanisms featuring miRNAs that regulate utrophin expression, and demonstrated that these mechanisms can be targeted to increase endogenous utrophin expression in cultured muscle cells. We suggest that miRNA-mediated inhibitory mechanisms could be targeted by methods similar to those described here as a novel strategy to increase utrophin expression as a therapy for DMD.

## Introduction

Duchenne muscular dystrophy (DMD) is an X-linked fatal neuromuscular disease caused by mutations in the dystrophin gene [1], [2]. Utrophin, a chromosome 6 encoded protein, bears significant homology and can functionally substitute for dystrophin when expressed at high levels [3], [4], [5]. High levels of utrophin expression in muscle occur during fetal development, when it is expressed throughout the sarcolemma. However, its expression declines leading to its spatially restricted expression at neuromuscular and myotendinous junctions of the adult myofiber sarcolemma. Indeed this developmental downregulation has been suggested as a reason for the delayed onset of muscle weakness in DMD [5], [6]. Direct evidence for the ability of utrophin to functionally compensate for dystrophin deficiency comes from experimental studies in animal models of DMD demonstrating that utrophin over-expression driven by transgenic means, viral vectors, pharmacological promoter activation or protein transduction can rescue dystrophin-deficient muscle in mice and dogs [7], [8], [9], [10], [11], [12], [13]. While promising, these strategies are still experimental and face considerable technical hurdles. A better understanding of the molecular events regulating utrophin expression is crucial in order to facilitate the development of strategies aimed at upregulation of utrophin in muscle fibers of DMD patients.

Utrophin expression is driven by two different promoters, namely A and B [14], [15], although myofiber expression is predominantly driven by promoter A. Some of the regulatory mechanisms playing major roles during transcription via the utrophin-A promoter have been identified [16], [17], [18], [19] and numerous studies have focused on this promoter to model its expression and achieve upregulation [11], [12], [13], [20], [21], [22], [23], [24]. However, it is becoming increasingly evident that the regulation of utrophin expression is more complex than previously appreciated. For example, alongside *ets*-mediated utrophin-A promoter activation at the synapse [16], [17], ERF-mediated transcriptional silencing is thought to regulate its concurrent extra-synaptic repression in myofibers [25]. Recently it has been shown that the expression of utrophin is modulated further at the level of translation. Utrophin-A and -B transcripts differ at their 5′-untranslated regions (5′-UTRs) and activation of an internal ribosome entry site (IRES) present at the utrophin-A 5′-UTR contributes to utrophin-A upregulation during muscle regeneration and glucocorticoid treatment [26], [27]. The utrophin 3′-untranslated region (3′-UTR), on the other hand, has been shown to be the target of the miRNA miR-206 [28].

Given the large size of the utrophin 3′-UTR (2.4 kb), we hypothesized that it was likely to be targeted by more than one miRNA. Therefore, we used bioinformatics to predict miRNAs that would target the utrophin 3′-UTR, and validated the predictions using quantitative real-time PCR assays to confirm expression of the miRNAs in muscle, as well as transfections with exogenous pre-miRNAs to confirm targeting of the utrophin 3′-UTR. We used ribosomal profiling and luciferase reporter constructs to demonstrate that, in line with substantial targeting by miRNAs, the utrophin-A mRNA exists in a state of translational repression. Finally, we demonstrated that by inhibiting the actions of miRNAs, using miRNA binding site-blocking oligomers, we could de-repress the utrophin 3′-UTR and upregulate translation of endogenous utrophin protein. We suggest that inhibition of translation by miRNAs is an important mechanism regulating utrophin expression and that it could be targeted as a therapeutic strategy to upregulate utrophin in the myofiber in DMD.

## Results

### Bioinformatic predictions of miRNAs targeting the utrophin 3′-UTR

**Figure 1:**
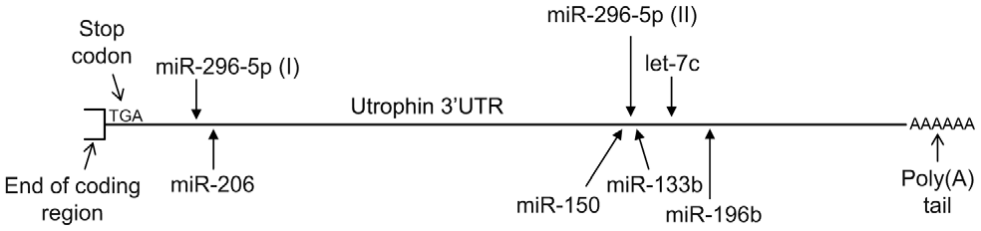
Six miRNAs are predicted to target the utrophin 3′-UTR. The miRanda v1.0b algorithm was used to predict miRNAs that target the utrophin 3′-UTR. Six miRNAs (miR-296-5p, miR-206, miR-150, miR-133b, let-7c, and miR-196b) were strong candidates and their predicted target sites within the utrophin 3′-UTR are represented diagrammatically. Note that miR-296-5p has two putative binding sites, as shown.

To date, miR-206 is the only miRNA that has been reported to target utrophin [28]. However, the very large size (2.4 kb) of the utrophin 3′-UTR suggests it could be a target of several regulatory miRNAs. Therefore, we used the miRanda v1.0b algorithm [29] to predict miRNAs that target the utrophin 3′-UTR, based on sequence and thermodynamic properties. Five additional miRNAs were found to be excellent candidates for targeting the mouse utrophin mRNA, namely, let-7c, miR-150, miR-196b, miR-296-5p, miR-133b and the previously reported miR-206. Interestingly, miR-133b and miR-206 are known to be muscle specific. These miRNAs are also predicted to target human utrophin. The positions of their predicted target sites in the utrophin 3′-UTR are shown in Fig. 1.

### The utrophin-A mRNA is translationally repressed

**Figure 2:**
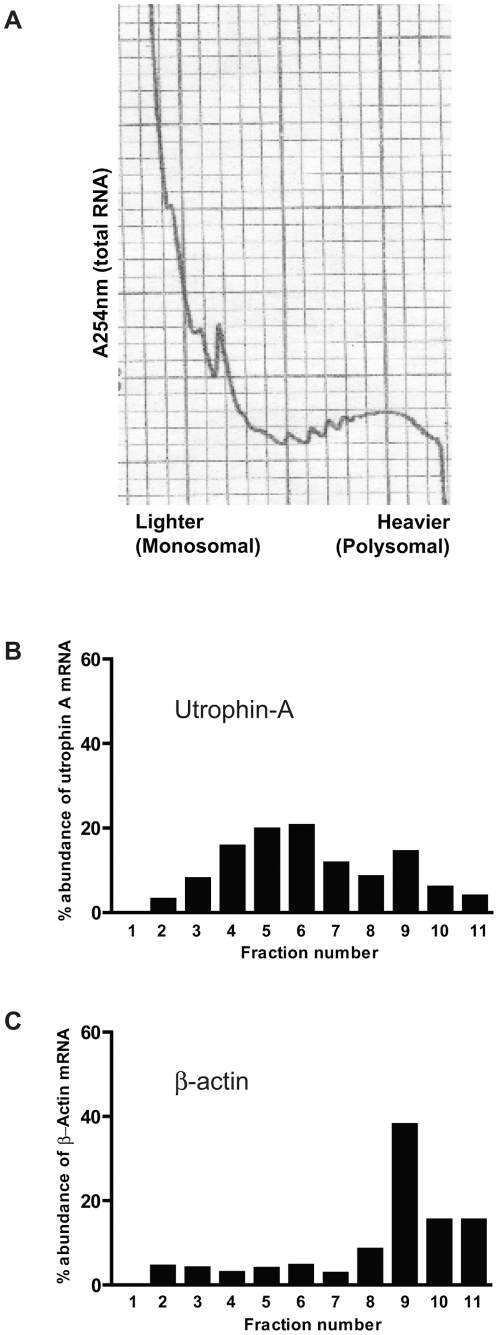
The utrophin-A mRNA is translationally repressed. Ribosomal profiling of utrophin-A and β-actin mRNAs from C2C12 cells after sucrose density gradient fractionation. (A) Typical ribosomal profiling trace, showing total RNA abundance across fractions as measured by absorbance at 254 nm. (B–C) While the utrophin-A mRNA is found in lighter, monosomal fractions (B), that of β-actin is enriched in heavier, polysomal fractions (C), showing the lower translation initiation efficiency of utrophin-A mRNA in C2C12 cells. A254, absorbance at 254 nm.

To validate the prediction that the utrophin-A mRNA is targeted by multiple miRNAs, we first used ribosomal profiling of the utrophin-A mRNA in mouse myoblast C2C12 cells to determine whether it is translationally repressed. As shown in Fig. 2, the utrophin-A mRNA is found in lighter, monosomal fractions, indicating that it is associated with one or only a few ribosomes and is therefore being translated inefficiently. In contrast, the β-actin mRNA is found in heavier, polysomal fractions, indicating that it is associated with many ribosomes. Therefore, in C2C12 cells, the utrophin-A mRNA exists in a state of translational repression.

### The 5′- and 3′-UTRs of utrophin mediate translational repression

**Figure 3:**
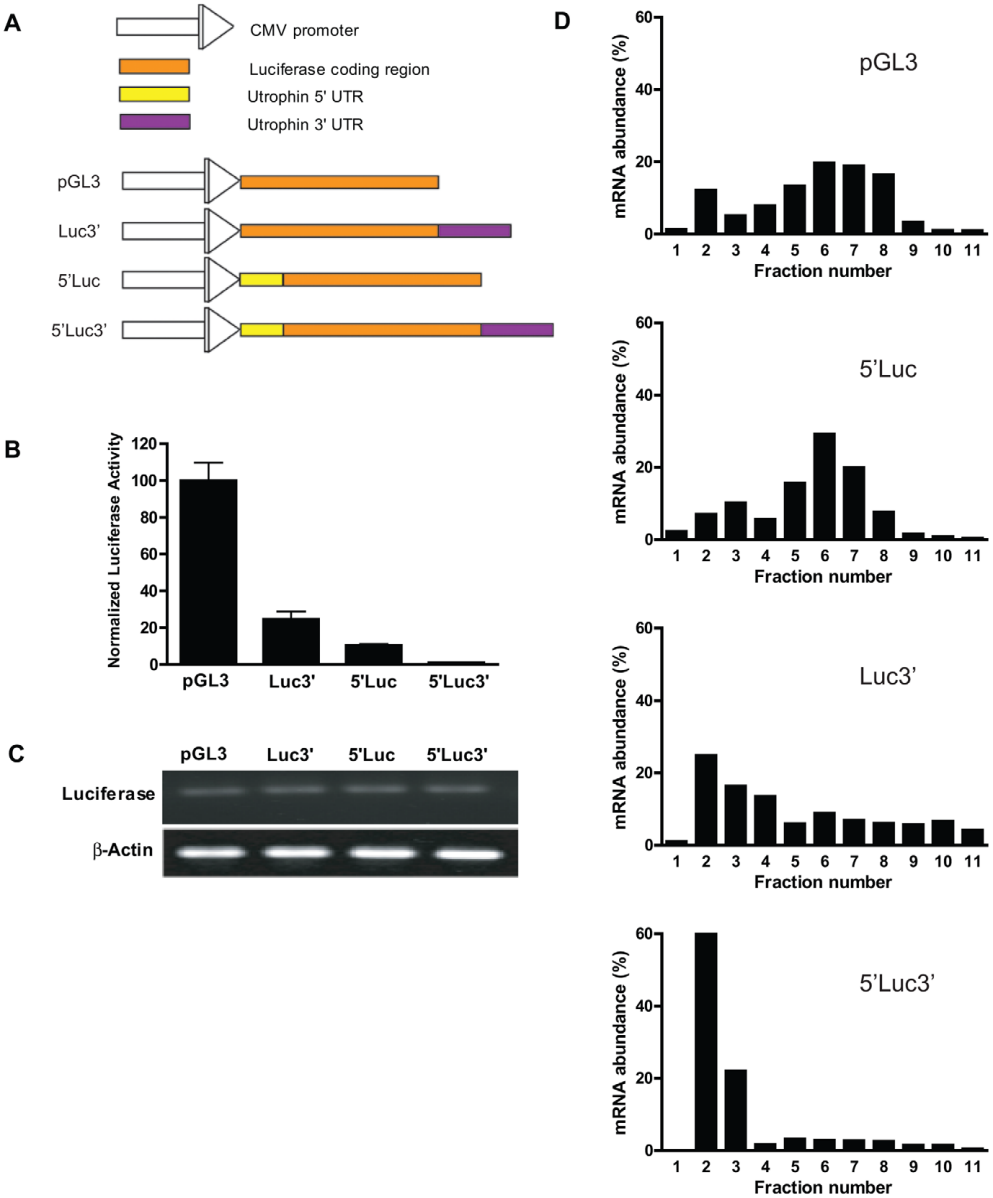
The 5′- and 3′-UTRs of utrophin-A mediate translational repression. (A) Schematic of firefly luciferase reporter constructs, pGL3, Luc3′, 5′Luc and 5′Luc3′. All constructs are derivatives of pGL3 and under control of the CMV promoter. In Luc3′, the luciferase coding sequence is appended with the 3′-UTR of utrophin, in 5′Luc, the 5′-UTR of utrophin-A is inserted just before the start codon of luciferase and in 5′Luc3′ the reporter is flanked by the 5′- and 3′-UTRs of utrophin-A. (B) C2C12 cells were transfected with the four constructs in equimolar amounts, and luciferase activity measured 6 hours post-transfection. Firefly luciferase activity normalized to Renilla luciferase from the co-transfected endogenous control pRL-TK is shown. Bars represent mean ± standard deviation (SD) from six independent experiments. (C) RNA was extracted from parallel samples and expression of firefly luciferase and β-actin studied by RT-PCR, showing that the transcriptional activity of the four constructs is the same. (D) C2C12 cells were transfected with pGL3, 3′Luc, 5′Luc or 5′Luc3′ in equimolar amounts. Lysates were harvested 24 hours post-transfection and subjected to ribosomal profiling. The distribution of luciferase transcripts from each construct was determined by real time PCR.

As both the 3′- and 5′-UTRs of genes are known to mediate miRNA-based repression, we made four reporter constructs, based on the pGL3 vector (Fig. 3A), to determine the contributions of the 5′- and 3′-UTRs of the utrophin mRNA towards its translational repression. In 5′Luc, the 5′-UTR of the utrophin-A mRNA was cloned upstream of the luciferase coding region. The 3′-UTR was cloned downstream of the luciferase coding region to obtain Luc3′. In 5′Luc3′, the luciferase coding region is flanked by the 5′- and 3′-UTRs of the utrophin-A mRNA.

Equimolar amounts of these constructs were transfected into C2C12 cells and luciferase activity was assayed. The addition of the 5′- or 3′-UTR reduced luciferase activity by ∼92% or ∼80% respectively, compared to the parent construct pGL3. The addition of both UTRs decreased luciferase activity by ∼99%; an amount greater than each element alone suggesting co-operability between these elements (Fig. 3B). RT-PCR confirmed that there was no difference in mRNA levels produced by the four constructs (Fig. 3C), demonstrating that the inhibition was at the level of translation. Similar results were observed when HeLa cells were transfected with these constructs (Fig. S1), suggesting that this mechanism is not limited to C2C12 cells.

To determine how the 5′- and 3′-UTRs contribute to the inefficient association of the utrophin-A mRNA with ribosomes, we performed ribosomal profiling on C2C12 cells transfected with the four reporter constructs described above. Compared to pGL3, the Luc3′ mRNA was shifted towards lighter, less efficiently translating fractions (Fig. 3; compare A and C). An even greater shift was observed for 5′Luc3′ (Fig. 3D); ∼80% of the mRNA was present in fractions 2 and 3, which represent mostly translationally inactive, non-polysomal ribosomes. This suggests that the 3′-UTR of the utrophin mRNA causes a reduction in its ribosomal association, and that this effect is exacerbated in the presence of the 5′-UTR.

### miRNAs contribute to 3′-UTR-mediated repression of utrophin translation

**Figure 4:**
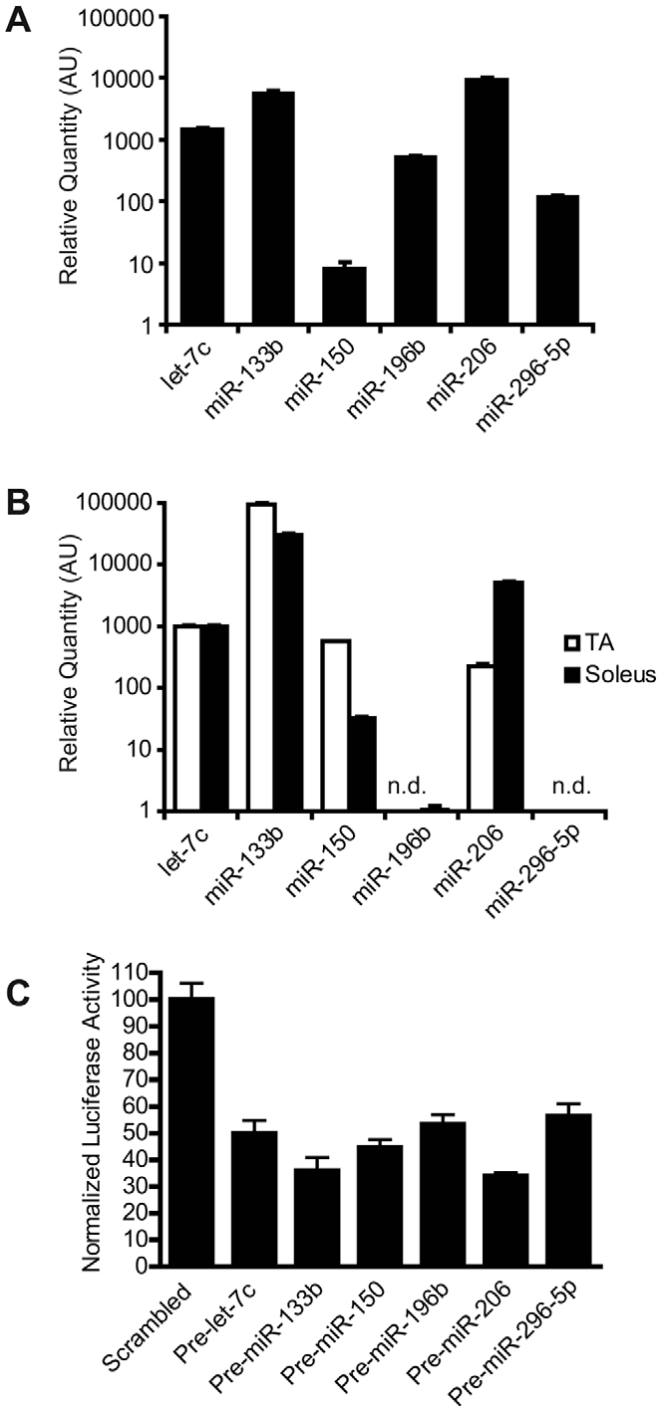
Predicted miRNAs are expressed in C2C12 cells and skeletal muscle and can target the utrophin 3′-UTR. Expression levels of miRNAs in C2C12 cells (A) and TA and soleus muscles (B) were quantified using TaqMan miRNA assays. All six miRNAs are expressed in C2C12 cells. miR-150 was not detected (n.d.) in TA, while miR-296-5p was n.d. in TA or soleus. Bars represent mean ± SD. (C) HeLa cells, which do not express miR-206, were transfected with 5′Luc3′ and pRL-TK with different pre-miRNAs precursors or a scrambled negative control. Cells were harvested 6 hours post-transfection and a luciferase assay performed. Firefly luciferase activities were normalized to pRL-TK derived Renilla luciferase activity and expressed as percentage normalized luciferase activity of the negative control transfected cells. Normalized luciferase activity decreases in every pre-miRNA transfected set. Bars represent mean ± SD from six independent experiments.

To validate the bioinformatic predictions and determine whether miRNAs are responsible for the translational repression mediated by the utrophin 3′-UTR, we first verified whether the six miRNAs were expressed in cultured C2C12 myoblasts and fast and slow skeletal muscles of mice. All six miRNAs were expressed in C2C12 cells and expressed at varying levels in muscle (Fig. 4A and B).

Next, we co-transfected pre-miRNAs (miRNA precursor RNA stem-loops) for each of the miRNAs of interest or a scrambled pre-miRNA control, with the 5′Luc3′ construct, in which the 3′- and 5′-UTRs of utrophin flank the coding sequence for firefly luciferase, into cultured HeLa cells. The Renilla luciferase expression plasmid pRL-TK was used as a control for transfection efficiency. HeLa cells were selected because they do not express endogenous miR-206, which might mask the effects (if any) of miR-206 added exogenously. A luciferase assay was performed 6 hours post-transfection. Compared to the scrambled control, all the pre-miRNAs tested produced a reduction in luciferase activity, confirming that all six miRNAs can target the utrophin 3′-UTR and repress translation (Fig. 4C).

### miRNA inhibition relieves utrophin 3′-UTR-mediated translational repression

**Figure 5:**
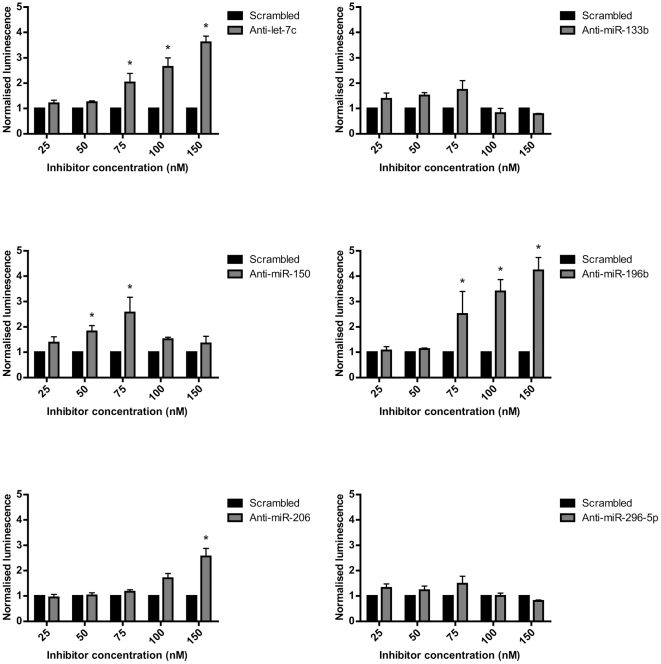
miRNA inhibition can de-repress the utrophin 3′-UTR and upregulate translation. C2C12 cells were transfected with 5′Luc3′, pRL-TK and different antisense miRNA inhibitors or a scrambled control inhibitor, at a range of concentrations. Luciferase assays were performed 24 hours post-transfection. Firefly/Renilla ratios in the presence of miRNA inhibitors were normalized to ratios in the presence of a scrambled inhibitor. Inhibitors of let-7, miR-150, miR-196b and miR-206 increased normalized luciferase activity, whereas inhibitors of miR-133b and miR-296-5p did not produce any significant upregulation. Bars represent mean ± standard error from three independent experiments. * Significantly different from scrambled inhibitor by two-way ANOVA followed by Bonferroni posttests, p<0.05.

Having demonstrated that six miRNAs target the utrophin 3′-UTR, we asked whether inhibition of these miRNAs could de-repress the 3′-UTR and upregulate translation. Therefore, C2C12 cells were transfected with 5′Luc3′ together with antisense inhibitors of the six utrophin-targeting miRNAs. Luciferase activity was assayed 24 hours post-transfection. As shown in Fig. 5, inhibitors of let-7c, miR-150, miR-196b and miR-206 were able to de-repress the utrophin 3′-UTR in a dose-dependent manner and produce increases of up to 4-fold in luciferase translation.

**Figure 6:**
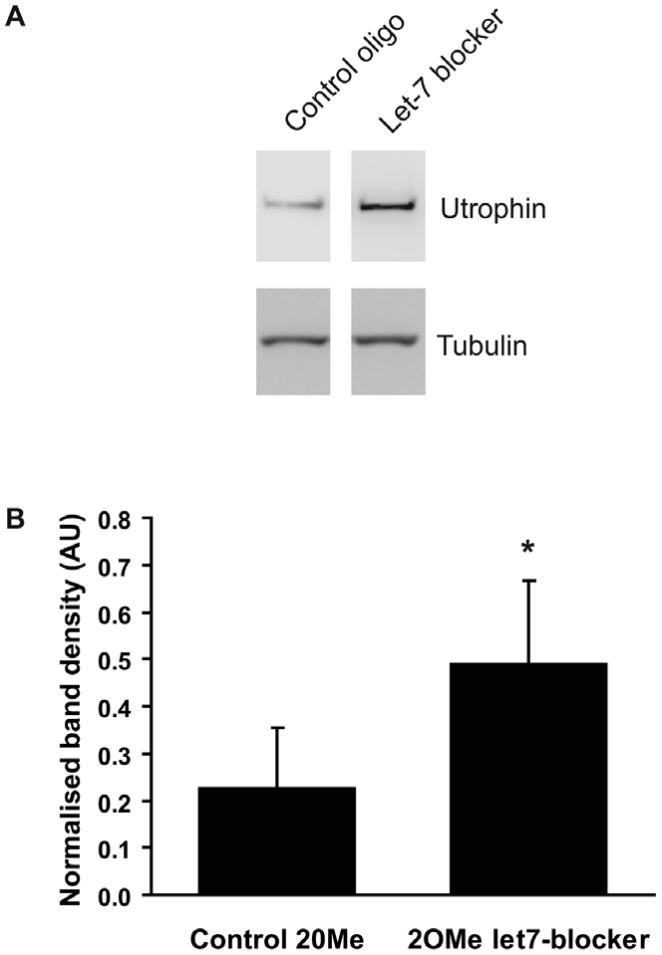
2OMePS let7-blocker upregulates endogenous utrophin protein. C2C12 cells were transfected with 300 nM control or let7-blocking 2OMePS oligomers. (Note DMSO (0.025%) was also present in both cases). Endogenous utrophin protein was assayed by Western blotting after 72 hours. A. Representative Western blot. B. Quantification of utrophin band density normalized to tubulin band density. Bars represent mean ± standard error from 3 independent experiments. The let7-blocker increased endogenous utrophin protein by 2.2-fold, compared to the control 2OMePS. * Significantly different from control 2OMePS by paired T test, p<0.05.

Next we wished to confirm that endogenous utrophin protein levels could be upregulated by inhibition of miRNAs. To do this, we used oligomers consisting of 2-O-methyl modified bases on a phosphorothioate backbone (2OMePOs). The 2OMePOs were designed to bind to the utrophin 3′UTR and block the let-7 family target site situated therein (Fig. 1), thus preventing utrophin translational repression by let-7c or other let-7 family members. This strategy should, in principle, be relatively specific for utrophin, rather than affecting other let-7 target genes. We used the 2OMePS chemistry because these oligomers are suitable for *in vivo* delivery and can be synthesized on a larger scale. Additionally, in our hands 2OMePSs had lower cytotoxicity than the commercially available miRNA inhibitors. C2C12 cells were transfected with either a let-7-blocking 2OMePS or an inactivate control 2OMePS. DMSO (0.025%) was present in both cases due to co-testing of other substances. Cell lysates were harvested after 72 hours and levels of utrophin protein measured by Western blotting. As shown in Fig. 6, treatment with the let-7-blocking 2OMePS oligomer increased endogenous protein levels by over 2-fold, compared to the inactive control 2OMePS oligomer. This demonstrates that endogenous utrophin protein levels can be increased by blocking the actions of miRNAs, and validates the concept that miRNA inhibition could be used to upregulate utrophin, as a potential therapy for DMD.

## Discussion

In this study, we used a variety of bioinformatic, molecular and cell biological methods to demonstrate the role of miRNAs in the post-transcriptional control of utrophin expression. We show that at least six miRNAs target the utrophin 3′-UTR. We also demonstrate that inhibition of utrophin-targeting miRNAs can de-repress the utrophin 3′-UTR, leading to an upregulation of utrophin protein expression. We suggest that these mechanisms could be targeted to upregulate utrophin in DMD.

Previous work has shown that the utrophin 5′-UTR inhibits cap-dependent translation in muscle, probably due to its predicted complex secondary structure, but that an IRES located in the 5′-UTR is activated during muscle regeneration and in proliferating C2C12 myoblasts [26]. In our study, we confirm the inhibitory effect of the utrophin 5′-UTR in C2C12 cells. Interestingly, we find that the 5′- and 3′-UTRs can act synergistically, such that each potentiates the inhibition caused by the other. Our ribosomal profiling experiments using reporter constructs shed light on the mechanisms of inhibition by the 5′- and 3′-UTRs, suggesting that they cause an inhibition of translational initiation from the utrophin-A mRNA, thus limiting ribosome occupancy.

Having shown the importance of the 3′-UTR in repressing utrophin translation, and its interaction with the 5′-UTR, we demonstrated that this repression is mediated, at least in part, by miRNAs. We identified five new miRNAs (let-7c, miR-150, miR-196b, miR-296-5p and miR-133b) that target the utrophin 3′-UTR and confirmed the previously reported targeting by miR-206 [28]. Importantly from a therapeutic point of view, these six miRNAs are also predicted to target utrophin in humans.

We tested whether antisense inhibition of these miRNAs could upregulate utrophin expression, and achieved this for four of the six miRNAs tested. It is not yet clear why inhibition of the other two miRNAs did not have the same effect. However, in addition to issues related to stability/chemistry of the inhibitors, some of the targeted miRNAs are only present at low levels in C2C12 cells, and therefore decreasing their expression would be predicted to have little effect on reporter construct expression.

Of the miRNAs studied, let-7c stood out as the best initial target as it is highly expressed in fast and slow skeletal muscles, and its antisense inhibition in C2C12 cells caused a 4-fold translational upregulation of the luciferase reporter. For this reason, we focused on let-7c for further experiments, and showed that blocking the binding of let-7 family miRNAs to the utrophin 3′-UTR, using a 2OMePO oligomer, could upregulate endogenous utrophin protein by over 2-fold, in C2C12 cells. The difference in the degree of response between the two experiments could be due to different time points examined (the utrophin gene is much larger than luciferase so more time was allowed to see a change in protein levels) or different amounts of transfection reagent used. Importantly, our results demonstrate that inhibition of miRNAs can de-repress the utrophin 3′-UTR and upregulate translation of utrophin protein, making it a viable therapeutic strategy for DMD.

For experiments investigating endogenous utrophin protein, we used 2OMePO oligomers designed to bind the utrophin 3′UTR and block the actions of let-7 family miRNAs, in place of commercially produced antisense miRNA inhibitors. The success of these experiments is greatly encouraging, given that 2OMePO oligomers can be synthesized on a relatively large scale are suitable for use *in vivo*. Indeed, this chemistry has been used in clinical trials of exon-skipping therapeutics in patients with DMD [30], [31]. Furthermore, targeting the miRNA binding site in the utrophin 3′-UTR is likely to be more specific than targeting the miRNA itself, given that any one miRNA typically targets a number of different mRNAs.

Although our results demonstrate a crucial role of miRNAs in 3′-UTR-mediated inhibition of IRES initiation they do not exclude the possibility of other mechanisms being in operation. For example, it has recently been found that an AU-rich element in the 3′-UTR modulates utrophin mRNA stability [32].

In conclusion, we have shown that the utrophin-A mRNA is subject to a significant degree of translational repression, mediated by its 5′- and 3′-UTRs, and that the actions of miRNAs contribute significantly to this repression. We identify five novel miRNAs that target the utrophin 3′-UTR and demonstrate that inhibition of miRNA targeting can de-repress the utrophin 3′-UTR, leading to an upregulation in utrophin protein translation. Therefore, we believe that utrophin upregulation by miRNA inhibition represents a novel therapeutic strategy for DMD.

## Materials and Methods

### miRNA prediction

miRNAs targeting the utrophin 3′-UTR were predicted using the miRanda v1.0b algorithm, with a cut-off for predictions of a score greater than 100 and minimum free energy of -14 kcal/mol.

### Constructs

The mouse utrophin-A 5′-UTR was amplified with primers 5′CCATGGGATCCACGGCTCCGAGG3′ and 5′CCATGGCTTGAATGAGTTTCAG TATAATCCAAAG3′ and cloned upstream of luciferase at the NcoI site of pGL3 to construct 5′Luc. Cloning of the 5′-UTR at the NcoI site converted the poor Kozak consensus of aagATGG for native utrophin-A into a good Kozak consensus of gccATGG. pGL3 also contains a good Kozak sequence, accATGG. A ScaI-BamHI digested fragment of Riken clone 9430078L05 (NCBI Accession # AK035043) containing the mouse utrophin 3′-UTR was cloned at the XbaI site of pGL3 and 5′Luc by blunt end ligation to construct Luc3′ and 5′Luc3′, respectively. The final constructs contain the full 2.4 kb mouse utrophin 3′-UTR preceded by the final 200 bases of utrophin coding sequence.

### Cell culture

The mouse muscle C2C12 and human HeLa cell lines (both from ATCC) were cultured in DMEM with 10% FBS, glutamine, penicillin and streptomycin.

### Transfection

All transfections were done with Lipofectamine 2000 (Invitrogen) according to the manufacturer's protocol. For 2OMePS transfections, the ratio was reduced to 1∶1 µl Lipofectamine 2000:µg oligomer.

### Ribosomal profiling

C2C12 cells (70% confluent) were transfected with constructs in 100 mm dishes. Media was changed after 6 hours. Cycloheximide (final concentration 100 µg/ml) was added 24 post-transfection and incubated for 15 minutes at 37°C. Cells were washed twice with ice cold PBS, lysed in 300 µl ice cold lysis buffer (110 mM potassium acetate, 2 mM magnesium acetate, 10 mM HEPES [pH 7.5], 50 mM potassium chloride, 10 mM magnesium chloride, 2 mM DTT, 1% NP-40, 1% deoxycholate, complete-mini protease inhibitors (Roche), 500 U/ml RNasin, and 100 µg/ml cycloheximide), scraped into a tube, homogenized by passing 8 times through a 23 gauge needle at 4°C and centrifuged (10 minutes, 14000 rpm). Supernatants were layered onto 11 ml of a 15–50% linear sucrose gradient and centrifuged (36000 rpm, 2 hours). Gradients were fractionated by upward displacement with 60% sucrose and absorbance monitored continuously at 254 nm. RNA was isolated from each fraction with Trizol (Invitrogen) and treated with DNaseTURBO (Ambion) followed by treatment with DNase-*free* (Ambion). RNA was reverse transcribed with random hexamers using the SuperScript First Strand Synthesis System (Invitrogen), according to the manufacturer's instructions. Utrophin-A mRNA copy number was quantified from 10 µl cDNA with 0.05 nM each of primers 5′ATCCATTTGGTAAAGGTTTTCTTCTG3′ and 5′ACGAATTCAGTGAC ATCATTAAGTCC3′ and Tamra-labeled 5′ATCATTGTGTTCATCAGATC3′ MGB probe (0.25 µM) in TaqMan mix (Applied Biosystems). A standard curve was generated from dilutions of a clone containing a unique region of the utrophin-A cDNA, amplified with primers 5′GCGTGCAGTGGACCATTTTTCAGATTTA3′ and 5′GCGTGCA GATCGAGCGTTTATCCATTTG3′. β-actin was quantified using pre-mixed reagents (Ambion). A standard curve was generated from dilutions of a β-actin cDNA clone amplified with primers 5′TTCTTTGCAGCTCCTTCGTTG3′ and 5′TCAAGTCAG TGTACAGGCCAGC3′. Luciferase transcript levels in transfected cells were determined by SYBR Green qPCR using 5 µl cDNA with 10 pmol primers 5′AAAGTTGCGCGGAGGAGTT3′ and 5′CCCTTCTTGGCCTTTATGAGG3′ (firefly luciferase) or 5′ATCGGACCCAGGATT CTTTTC3′ and 5′CCATTTCATCAGGTGCATCT3′ (Renilla luciferase) in SYBR Green PCR mix (Applied Biosystems). A standard curve was generated using dilutions of pGL3 (firefly luciferase) or pRL-TK (Renilla).

### Luciferase reporter assay

C2C12 cells were plated in 24 well plates, 40,000 cells per well, 1 day before transfection. 400 ng pGL3 (1600 ng for 6-well plates) or equimolar amounts of other constructs were transfected, with 50 ng pRL-TK (Promega), per well. Reporter activity was measured by Dual Luciferase Assay (Promega) 6 or 24 hours after transfection.

### RT-PCR for luciferase and β-actin

RNA was isolated using an RNeasy kit (Qiagen) and reverse-transcribed with random hexamers using the SuperScript First Strand Synthesis System (Invitrogen), according to the manufacturer's instructions. PCR amplification was done using primers 5′AAAGTTGCGCGG AGGAGTT3′ and 5′CCCTTCTTGGCCTTTATGAGG3′ for luciferase or 5′CGTGCGTGACATCAAAGAGAAGC3′ and 5′CCCAAGAAGGAAGGCTGGAA AAG3′ for β-actin.

### Pre-miRNAs and miRNA inhibitors

Pre-miRNAs or miRNA antisense inhibitors (Ambion) were transfected into C2C12 cells with 680 ng 5′Luc3′ and 50 ng pRL-TK per well in 24-well plates. Pre-miRNAs for hsa-let-7c (PM10436), hsa-miR-133b (PM10029), hsa-miR-150 (PM10070), hsa-miR-196b (PM12946), hsa-miR-206 (PM10409) and hsa-miR-295-5p (PM10609) were used, with a scrambled pre-miRNA (pre-miRNA negative control #1). Inhibitors of hsa-let-7c (AM10436), hsa-miR-133b (AM10029), hsa-miR-150 (AM10070), hsa-miR-196b (AM12946), hsa-miR-206 (AM10409) and hsa-miR-295-5p (AM10609) were used, all targeting both human and mouse miRNAs, or scrambled inhibitor anti-miR negative control #1. The 2OMePS oligomer designed to block the let-7 target site in the utrophin 3′-UTR had the sequence CUGAGGUAGAAAGGUGAUCAUGGCUC while the inactive control 2OMePS had the sequence GUGAGCACUUCUUUCCUUCUUUUUU.

### miRNA isolation, reverse-transcription and TaqMan quantitative real-time PCR analysis

An RNeasy Plus Kit (Qiagen) and provided supplementary protocol and a miRVana kit (Ambion) were used to prepare total RNA, containing miRNA, from skeletal muscles (tibialis anterior (TA) and soleus) of adult Black10 mice and C2C12 cells, respectively. RNA quality was estimated with a NanoDrop ND-1000 Spectrometer (Thermo Scientific). 10 ng (skeletal muscles) or 320 ng (C2C12 cells) total RNA was converted to cDNA using TaqMan miRNA Assay primers and TaqMan miRNA Reverse Transcription Kit (both Applied Biosystems).

Targeted sequences:

let-7c UGAGGUAGUAGGUUGUAUGGUU

miR-133b UUGGUCCCCUUCAACCAGCUA

miR-150 UCUCCCAACCCUUGUACCAGUG

miR-196b UAGGUAGUUUCCUGUUGUUGG

miR-206 UGGAAUGUAAGGAAGUGUGUGG

miR-296-5p AGGGCCCCCCCUCAAUCCUGU

Quantitative PCR (qPCR) was performed on a ABI PRISM 7900HT Real-Time PCR system (Applied Biosystems), and data analyzed with SDS.2.3 software. Expression levels of miRNAs were normalized to the endogenous control RNU6 in skeletal muscle, and the endogenous control sno202 in C2C12 cells (both assays from Ambion).

### Western blotting

Cell lysates were prepared by scraping with TNEC lysis buffer (1.5 mM Tris-HCl pH 8, 2.15 mM NaCl, 3.1% Igepal CA630, 4.2 mM EDTA with Complete protease inhibitors (Roche)), incubating on ice for 20 minutes then centrifuging at 13 000 rpm in a benchtop centrifuge at 4°C and removing and retaining supernatants. Protein concentration was assayed using a DC protein assay (Bio-Rad). 60–65 µg protein were combined with LDS sample buffer and NuPAGE reducing reagent (both Invitrogen) and heated to 99°C in for 5 minutes, then separated on 3–8% Tris-Acetate gels (Invitrogen) with TA running buffer for 2 hours 15 minutes at 80 V. Proteins were transferred to PVDF membranes for overnight at 35 V, 4°C in ice-cooled transfer buffer (25 mM Tris pH 8.3, 192 mM glycine, 20% methanol, 0.05% sodium dodecyl sulphate). Membranes were blocked for 1 hour at room temperature in 5% non fat milk in TBS (50 mM Tris pH 7.5, 150 mM NaCl), then probed for utrophin (upper half of membrane) with mouse monoclonal anti-utrophin antibody mancho 3 clone 8A4 (developed by Glenn E. Morris and obtained from the Developmental Studies Hybridoma Bank developed under the auspices of the NICHD and maintained by The University of Iowa Department of Biology) diluted 1∶50 in 5% non fat milk in TBST (TBS with 0.05% Tween 20), or tubulin (lower half of membrane) with anti-alpha-tubulin antibody clone DM1A (Sigma) diluted 1∶5000 in 5% non fat milk in TBS, for 1 hour at room temperature. Membranes were washed in 3 changes of TBST for 10 minutes each, then incubated with HRP-conjugated goat-anti-mouse IgG (Jackson ImmunoResearch), diluted 1∶4000 in 5% non fat milk in TBS (for utrophin) or TBS (for utrophin), for 1 hour at room temperature. TBST washes were repeated, then bands were visualized using SuperSignal West Pico Chemiluminescent Substrate (Thermo Scientific) and images obtained using an LAS-3000 Imager (Fujifilm). For presentation clarity, images were then inverted to give dark bands on a light background. Band densities were quantified using ImageJ (http://rsbweb.nih.gov/ij/index.html).

## Supporting Information

Figure S1 **Utrophin translation is repressed by UTRs in HeLa cells.** HeLa cells were transfected with pGL3, Luc3′. 5′Luc and 5′Luc3′ in equimolar amounts along with pRL-TK, and luciferase activity was measured 6 hours post-transfection. Normalized luciferase activity from the constructs was plotted as percentage of pGL3 activity. Bars represent mean values ± SD from six independent experiments.
(TIF)
