# Supplementary figures and images for: Translational Regulation of Utrophin by miRNAs
Source: PLoS One. 2011 Dec 27;6(12):e29376. doi: 10.1371/journal.pone.0029376 (PMC3246502; doi:10.1371/journal.pone.0029376)

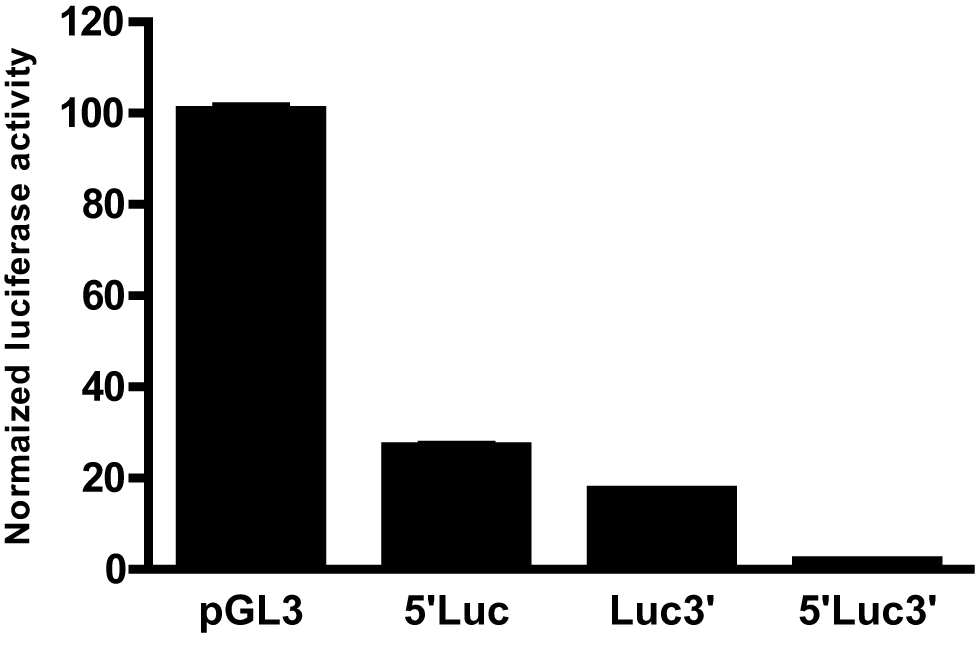

Supplement: Figure S1 — Utrophin translation is repressed by UTRs in HeLa cells. HeLa cells were transfected with pGL3, Luc3′. 5′Luc and 5′Luc3′ in equimolar amounts along with pRL-TK, and luciferase activity was measured 6 hours post-transfection. Normalized luciferase activity from the constructs was plotted as percentage of pGL3 activity. Bars represent mean values ± SD from six independent experiments. (TIF) [file pone.0029376.s001.tif]
